# Unveiling the influence pathways of the evolution of human-nature relationships in the Yangtze river delta urban agglomeration

**DOI:** 10.1016/j.isci.2026.114989

**Published:** 2026-02-10

**Authors:** Hua Zhu, Liang Gan, Qing Zhang, Ligang Xu

**Affiliations:** 1 School of Geomatics, Zhejiang University of Water Resources and Electric Power, Hangzhou 310018, China; 2 Institute of Watershed Remote Sensing and Sustainable Development, Zhejiang University of Water Resources and Electric Power, Hangzhou 310018, China; 3 State Key Laboratory of Lake and Watershed Science for Water Security, Nanjing Institute of Geography and Limnology, Chinese Academy of Sciences, Nanjing 211135, China; 4 Jiangxi Province Poyang Lake Water Conservancy Hub Construction Office, Nanchang 330009, China; 5 Poyang Lake Wetland Research Station, Nanjing Institute of Geography and Limnology, Chinese Academy of Sciences, Jiujiang 332899, China

**Keywords:** earth sciences, environmental science, geomatics, urban planning

## Abstract

Understanding the interactions between human activities and ecosystems is crucial to enhancing human well-being and adaptive governance. This study employs the human footprint index (HFI) and the ecosystem quality index (EQI), along with multivariate statistical methods, to analyze human-nature relationships and their driving mechanisms in the Yangtze river delta urban agglomeration (YRDUA). The results show a steady increase in HFI and a decline in EQI from 2000 to 2020 in the YRDUA. The ratio of coordinated to conflicting areas has remained stable at approximately 3:5. Over 95% of the coupling coordination degree (CCD) were classified as primarily or moderately coordinated. Vegetation cover and human activity intensity were the main positive drivers of CCD in coordinated and conflicting regions, respectively, while terrain and climate were the dominant negative drivers. These findings deepen our understanding of human-nature interactions in urbanized areas, offering valuable insights for targeted ecosystem restoration.

## Highlights

• Coordination-to-conflict area ratio in the YRDUA reaches 3:5
• Human-nature relationships in the YRDUA display a polarized trend
• Drivers of human-nature relationships were identified using RF and PLS-SEM models
• Human activities’ positive effects on human-nature relations exceed negative ones

## Graphical abstract

**Figure:**
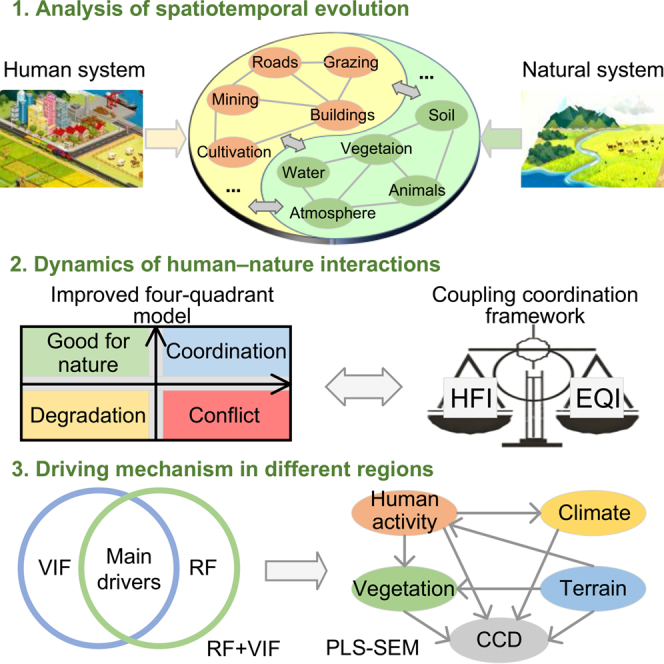

## Introduction

Urban areas, covering a mere 3% of the Earth’s surface yet housing nearly 50% of the global population and generating approximately 75% of the global economic output,^1^^,^^2^ stand out as a unique and pivotal force in driving economic and social progress. However, urban anthropogenic pressures have accelerated environmental degradation (e.g., pollution and biodiversity loss), via cascading effects (urban heat islands and climate change), ultimately compromising human well-being.^3^^,^^4^^,^^5^ International agreements and initiatives, including the COP15 Kunming-Montreal Global Biodiversity Framework and the IPBES “2030 Agenda,” have recognized cities as key areas for coordinating human activities with nature conservation.^6^^,^^7^ Urban agglomerations, as an advanced spatial configuration of integrated cities, are marked by intensified demographic concentration and land exploitation,^8^ thereby emerging as critical arenas of acute human-nature conflicts and intricate system interactions.^9^ Therefore, elucidating the interactions and key drivers of coupled human-natural systems within urban agglomerations is essential for mitigating ecological risks and advancing regional sustainability.

The human-nature relationship refers to the interactions and mutual influences between human activities and the natural environment. Research in this field focuses on exploring the symbiotic relationships and complex interactions between human social systems and natural environment systems.^3^^,^^10^^,^^11^ Numerous theories and models have been proposed to quantify these interactions, including system dynamics,^12^ the environmental Kuznets curve,^13^ and the coupling coordination degree (CCD) model.^14^ The increasing availability of fine-scale remote sensing data has enabled grid-based analyses of human and natural systems. However, most studies examine the relationship between specific human activities (e.g., urbanization, land use, etc.) and particular eco-environmental elements (e.g., biodiversity, vegetation cover, etc.) at various scales, including urban, metropolitan, provincial, and national levels.^15^^,^^16^^,^^17^^,^^18^^,^^19^ Few explore the relationship between human activities and the eco-environment as separate systems at the grid scale. In urban agglomerations, intensive human activities such as industrial production, transportation, and urban construction are intricately interwoven with diverse ecological components, including artificial grasslands, forests, and wetlands, forming a complex socio-ecological system that cannot be fully characterized by a single indicator.^6^^,^^20^^,^^21^ Recent studies have proposed frameworks for assessing human-nature interactions at fine spatial scales,^22^^,^^23^^,^^24^ but their application has been primarily limited to macro-regions, such as provincial,^23^ watershed,^25^ and national scales,^26^ which may not adequately reflect the high intensity and complexity of urban agglomerations. As a result, there remains a significant gap in the analysis of human-nature interactions within urban clusters, severely limiting our understanding of the underlying coupling mechanisms in regions with high human activity intensity.

Human-natural systems are shaped by a complex interplay of factors, including climate change, human activities, topography, and other multidimensional drivers.^24^^,^^27^ Understanding these interactions is crucial for uncovering the mechanisms of human-nature reciprocity and informing strategies that support sustainable development.^28^^,^^29^ Various methods have been employed to explore human-nature relationships, including the geodetector method for identifying key drivers of environmental outcomes, random forest (RF) as a machine learning approach for variable importance analysis, and generalized additive models for examining non-linear relationships.^23^^,^^27^ Although these approaches significantly enhance the capacity to explain and predict human-nature interactions,^30^^,^^31^^,^^32^ they remain limited in capturing the multidimensional and dynamic interactions across multiple spatial and temporal scales among various factors. This limitation restricts a comprehensive understanding of social-ecological systems in complex regions such as urban agglomerations.

Partial least squares structural equation modeling (PLS-SEM) has emerged as a robust alternative for analyzing multifactor causal relationships.^33^ Unlike traditional methods, PLS-SEM provides iterative estimates that optimize latent variable interpretation without requiring large sample sizes or normal data distributions.^34^ Its advantages have made it increasingly popular in ecological and environmental studies.^34^^,^^35^ Previous studies have used PLS-SEM to explore the driving mechanisms of human activities and ecological elements, such as urbanization,^36^ habitat quality,^37^ and vegetation dynamics.^38^ However, few have considered human-nature interactions as a coupled system, limiting the understanding of how human activity intensity influences ecological quality.

To sum up, existing studies primarily focus on the interaction between humans and natural systems at the provincial, watershed, and national scales,^23^^,^^25^^,^^26^ with little attention paid to the relationship between humans and natural systems within urban agglomerations, where interactions are more closely integrated. Although some studies have analyzed the interaction between urbanization and the ecological environment within urban agglomerations,^19^^,^^39^^,^^40^ these studies mostly concentrate on the relationship between specific human activities and ecological elements, lacking a comprehensive analysis of the overall human-nature relationship. Additionally, in the research on the human-nature relationship, current literature mainly examines the impact of single and dual factor interactions on this relationship,^23^^,^^26^^,^^27^ with little systematic understanding of the multi-factor interactions between driving forces and the human-nature system.

The Yangtze river delta urban agglomeration (YRDUA), China’s leading economic hub, provides a representative case for examining these dynamics. As one of the world’s largest and fastest-growing urban agglomerations, it offers insights that are broadly applicable to other rapidly urbanizing regions worldwide. The advantages of PLS-SEM in analyzing multi-factor interactions provide strong support for the analysis of driving mechanisms. Therefore, this study, using the YRDUA as the research area, applies PLS-SEM to reveal the direct, indirect, and interactive effects of influencing factors within human-natural systems, based on an analysis of the relationship between humans and nature. The study aims to answer the following questions: (1) what are the detailed patterns and synergistic evolution characteristics of the spatiotemporal dynamics between humans and natural systems within the complex spatial unit of urban agglomerations? (2) What are the dominant driving factors that shape the relationship between humans and natural systems under different human-nature relationship patterns? And, (3) through what direct, indirect paths, and interactions do these factors constitute the influencing mechanism? The findings not only advance scientific understanding of human-nature dynamics but also provide evidence-based guidance for differentiated governance, targeted ecosystem restoration, and sustainable urban development policies.

## Results

### Spatiotemporal characteristics of the human footprint index and the ecosystem quality index

**Figure 1:**
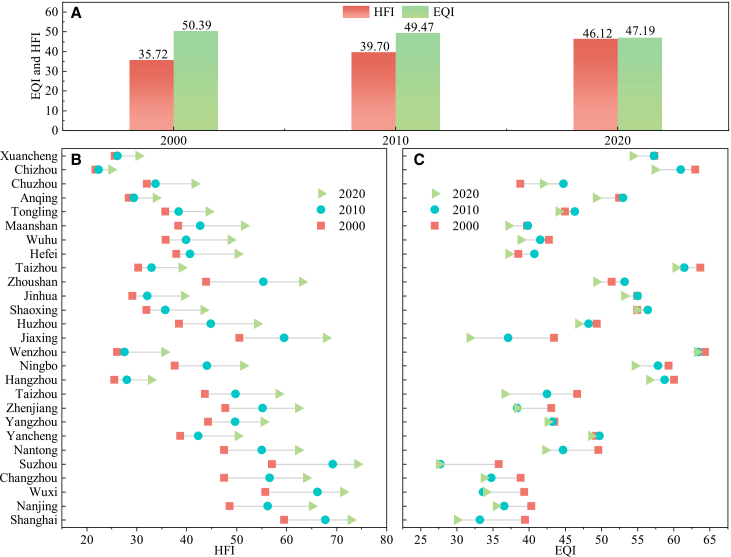
Mean value of HFI and EQI across YRDUA (A) Mean value of HFI and EQI in the YRDUA. (B) Average HFI of 27 cities in the YRDUA. (C) Average EQI of 27 cities in the YRDUA.

Figure 1 illustrates the mean change trends of the human footprint index (HFI) and the ecosystem quality index (EQI) in the YRDUA from 2000 to 2020. Overall, despite fluctuations, the changes in HFI and EQI do not exhibit significant inflection points and follow a linear trend (see Figure S1 in the supplemental information). The HFI increased from 35.72 to 46.12, whereas the EQI decreased from 50.39 to 47.19 (Figure 1A). At the city level, the mean HFI values generally followed the regional trajectory, showing consistent upward trends across most cities (Figure 1B). Similarly, changes in EQI were largely consistent with the overall decline, although nine cities (e.g., Yancheng, Jinhua, and Hefei) showed an initial increase followed by a decrease. For example, during this period, the EQI in Shanghai decreased from 39.44 to 27.48, while in Anqing, the EQI increased from 52.49 in 2000 to 52.99 in 2010, and then decreased to 49.19 in 2020. In contrast, Wuxi displayed the opposite trend, with EQI decreasing from 39.31 in 2000 to 33.63 in 2010, and then rising again to 33.99 in 2020 (Figure 1C). These results highlight a general intensification of human activities accompanied by ecosystem degradation across the region, with notable spatial heterogeneity in ecological responses.

**Figure 2:**
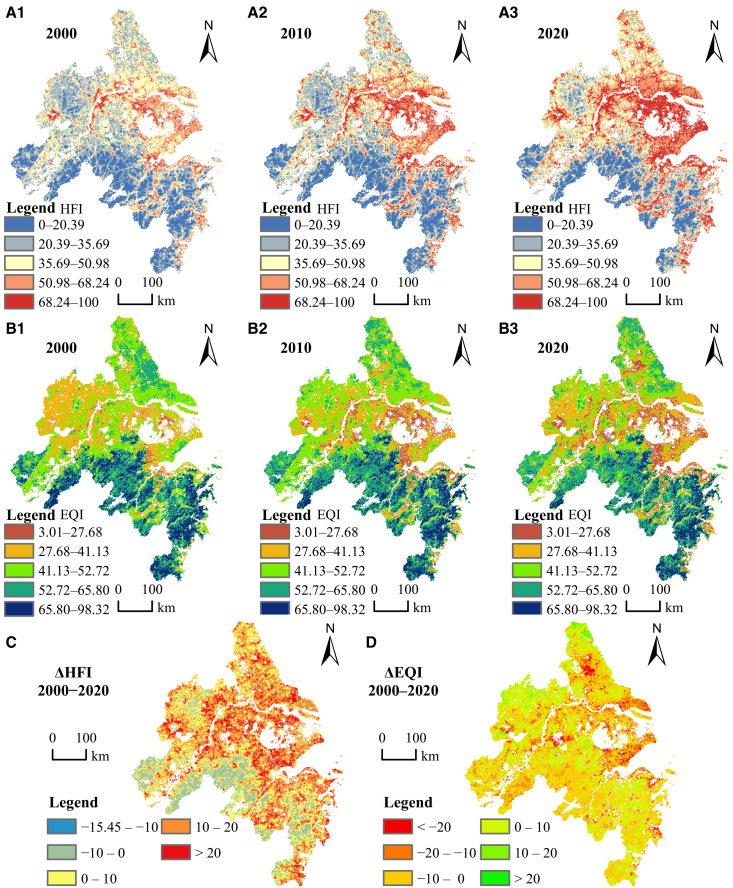
Spatiotemporal changing and trends of HFI and EQI (A1–A3) The spatial distribution of HFI from 2000 to 2020. (B1–B3) The spatial distribution of EQI from 2000 to 2020. (C) The difference in HFI between 2000 and 2020. (D) The difference in EQI between 2000 and 2020.

The HFI and EQI were each classified into five categories using the natural breaks method. Figure 2 shows their spatial distributions across the YRDUA from 2000 to 2020. The HFI exhibited a clear spatial gradient, declining from northeast to southwest (Figures 2A1–A3). High (50.98–100) and moderately high (35.69–50.98) values were concentrated in urban centers and surrounding areas, particularly around Taihu lake, along the Yangtze river, and in eastern coastal zones. Between 2000 and 2020, these regions expanded significantly, with increasing intensity, whereas areas of low HFI (0–20.39) were largely confined to the southern periphery of the YRDUA. Overall, HFI rose most sharply in urban regions, while slight decreases were observed in some rural areas (Figure 2C).

The EQI displayed a contrasting pattern, with the highest levels in the southwest, moderately high values in the north, and the lowest in the central urban belt (Figures 2B1–2B3). High EQI (52.72–98.32) was concentrated in Zhejiang and Anqing, with scattered areas in Yancheng and Nantong. Moderate EQI (41.13–52.72) occurred mainly in northern Jiangsu, while low EQI (3.01–41.13) dominated the urbanized areas around Taihu Lake and the Yangtze River corridor, including Shanghai, Wuxi, Nanjing, and Hefei. Over time, these low-value areas expanded, particularly around Taihu Lake, Taizhou, and parts of the Zhejiang coast, indicating progressive ecosystem degradation. Overall, the YRDUA showed predominantly slight decreases in EQI, with only limited areas experiencing slight improvement (Figure 2D).

### Identification of coordination and conflict zones

**Figure 3:**
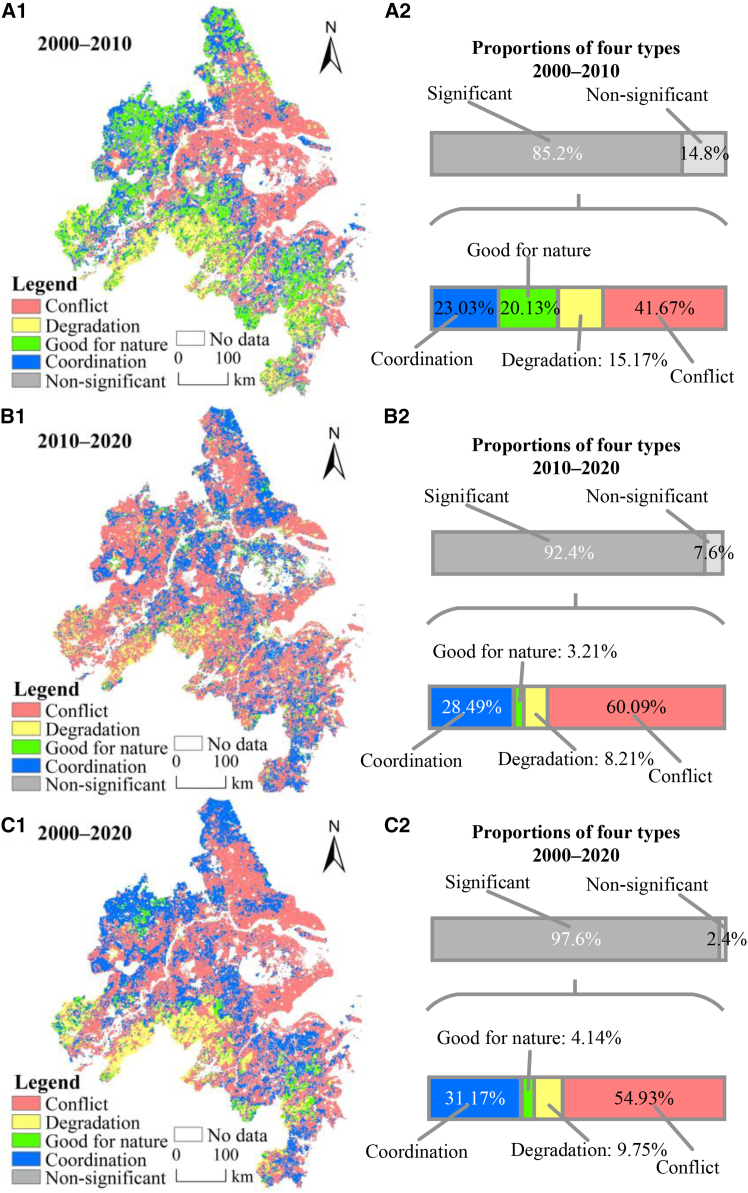
Dynamic relationships between HFI and EQI and proportions of four types of regions from 2000 to 2020 (A1 and A2) cover 2000–2010. (B1 and B2) cover 2010–2020. (C1 and C2) cover 2000–2020.

Based on the four-quadrant framework and the standard deviation threshold, regions with significant changes in HFI and EQI were identified for two periods: 2000–2010 and 2010–2020 (Figures 3A1 and 3B1). These regions accounted for 85.2% and 92.4% of the YRDUA, respectively (Figures 3A2 and 3B2). From 2000 to 2010, conflict areas dominated, covering 41.67% of the region and mainly distributed around Taihu Lake and along the Yangtze River. Coordinated areas accounted for 23.03%, primarily located in urban-rural fringes; good-for-nature regions covered 20.13%, concentrated in the forests of Zhejiang and cultivated land of Anhui; and degradation areas represented 15.17%, mainly on the southwestern periphery.

During 2010–2020, human-nature relationships exhibited a more polarized pattern (Figures 3C1 and 3C2). Conflict areas expanded substantially to 60.09% of the region, spreading across much of the YRDUA. Coordinated areas also increased to 28.49%, particularly in urban fringes and rural landscapes. In contrast, good-for-nature regions declined sharply to 3.21% and were scattered, while degradation areas accounted for 8.21%, retaining similar spatial distributions as in the previous decade.

Across the entire study period, the ratio of coordinated to conflicting regions was approximately 3:5 (Figure 3C2). Coordinated areas were mainly distributed in rural landscapes, while conflict zones remained concentrated around the Taihu lake and along the Yangtze liver. The intensification of human activities in these regions has contributed to ecological degradation, highlighting critical zones that require stronger policy intervention. Good-for-nature regions (4.14%) were concentrated in Taizhou, Jinhua, Anqing, and Chuzhou, whereas degradation areas (9.75%) were primarily found in Xuancheng and Chizhou. Overall, despite localized conflicts, the YRDUA has shown a gradual shift toward improved human-nature coordination over the past two decades.

### Coupling coordination level of the human footprint index and the ecosystem quality index

**Figure 4:**
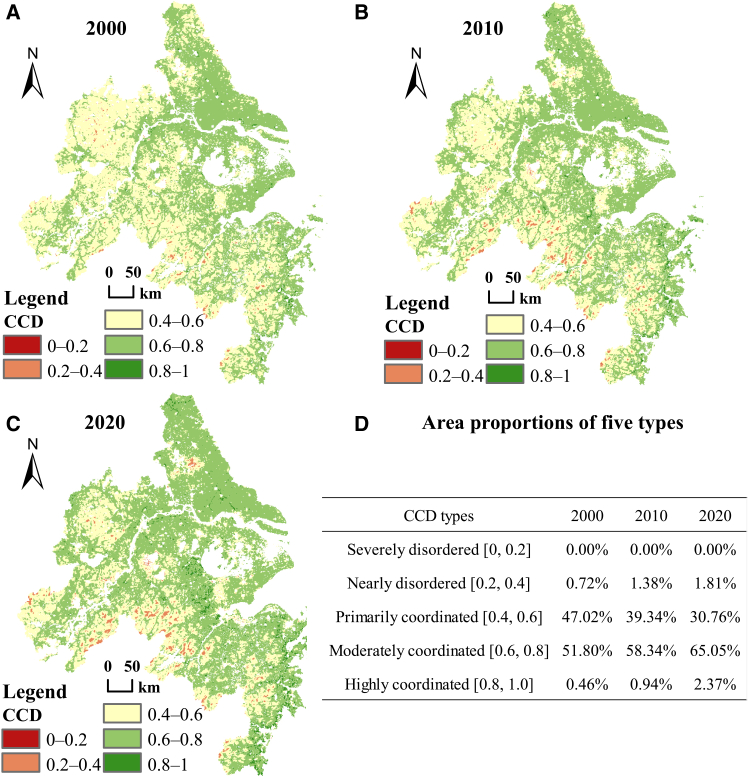
Spatiotemporal variation and area proportion of CCD between HFI and EQI (A–C) Spatial distribution of CCD in 2000, 2010 and 2020. (D) Area proportions of five CCD types.

As shown in Figure 4, the CCD between HFI and EQI in the YRDUA displayed pronounced spatiotemporal heterogeneity from 2000 to 2020. Overall, primarily and moderately coordinated types together accounted for more than 95% of the region. Primarily coordinated areas, concentrated in the southwestern YRDUA, gradually contracted toward the periphery, with their share decreasing from 47.02% in 2000 to 30.76% in 2020. In contrast, moderately coordinated areas, dominant in the northeast, expanded outward and increased from 51.80% to 65.05% over the same period. This trend reflects a gradual shift toward improved coordination between human activities and ecosystems. The evolution of this spatial pattern has closely aligned with the trajectory of ecological governance policies in the Yangtze river delta since 2000: from Shanghai’s “Three-Year Environmental Protection Action Plan” launched around 2000, through the promotion of regional collaborative governance of the ecological environment around 2010, to the elevation of ecological and green development as a core strategic priority under the Yangtze River Delta integration initiative in 2019.

Other CCD categories occupied only small proportions. Severely disordered areas were negligible across the study period. Nearly disordered areas, located mainly on the southwestern fringe, expanded modestly from 0.72% to 1.81%. Highly coordinated areas, scattered at city-landscape junctions, grew from 0.46% to 2.37%. These changes suggest that human-nature coordination in the YRDUA is evolving in a polarized manner, with moderate coordination becoming widespread while both nearly disordered and highly coordinated zones show localized growth.

### Dynamic relationships of the human footprint index and the ecosystem quality index in different types of regions

Three representative areas within the YRDUA—Pudong New Area (PNA, 121.544° E, 31.221° N), Suzhou Industrial Park (SIP, 120.723° E, 31.324° N), and a designated protection area (PA)—were selected to examine human-nature interactions under different development models.

**Figure 5:**
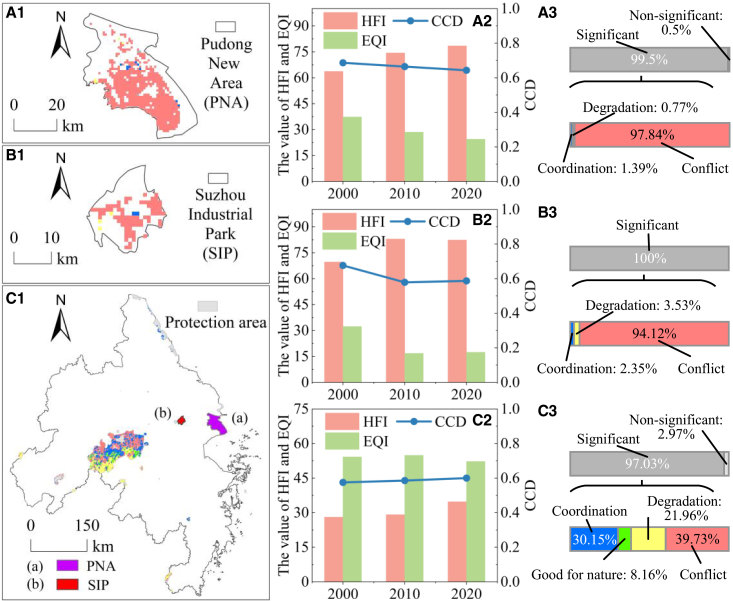
Relationship between HFI and EQI in key areas (A1–A3) PNA. (B1–B3) SIP. (C1–C3) PA. Note, the colors of the study area layers in subplots (A1), (B1), and (C1) are consistent with those in Figure 3.

The PNA (Figure 5A1), a core zone of high-intensity urbanization with dense land development and concentration of financial and technology industries, is subject to extreme human pressures. Between 2000 and 2020, the HFI rose steadily, while both EQI and CCD declined (Figure 5A2), indicating continuous deterioration in human-nature coordination. Conflict zones dominated (97.84%), with coordination and degradation together accounting for less than 3% (Figure 5A3), highlighting the ecological costs of megacity expansion.

In contrast, the SIP (Figure 5B1) illustrates a planning-guided model of industrial upgrading, integrating restructuring with strict environmental management. Here, HFI first increased and then declined, while EQI and CCD showed the opposite pattern (Figure 5B2). Conflict remained prevalent (94.12%), but its proportion was lower than in PNA, with coordination and degradation making up about 6% (Figure 5B3). Despite higher overall activity intensity, SIP achieved relatively better reconciliation between development and ecology, underscoring the role of scientific planning and resource efficiency in reducing conflicts.

The PA (Figure 5C1), serving as a habitat for rare and endangered species, exemplifies conservation-oriented management. Nearly all areas (97.03%) experienced significant changes, with HFI and CCD both increasing, while EQI first rose and later declined (Figure 5C2). Human-nature relationships were more balanced: coordination (30.15%) and conflict (39.73%) occurred in a 3:4 ratio (Figure 5C3), while degradation and good-for-nature together exceeded 30%. Compared with the YRDUA as a whole, the PA showed a much lower share of conflict (39.73% vs. 54.93%), demonstrating the effectiveness of conservation measures in sustaining ecological resilience.

### Screening of driving factors for the coupling coordination degree

**Figure 6:**
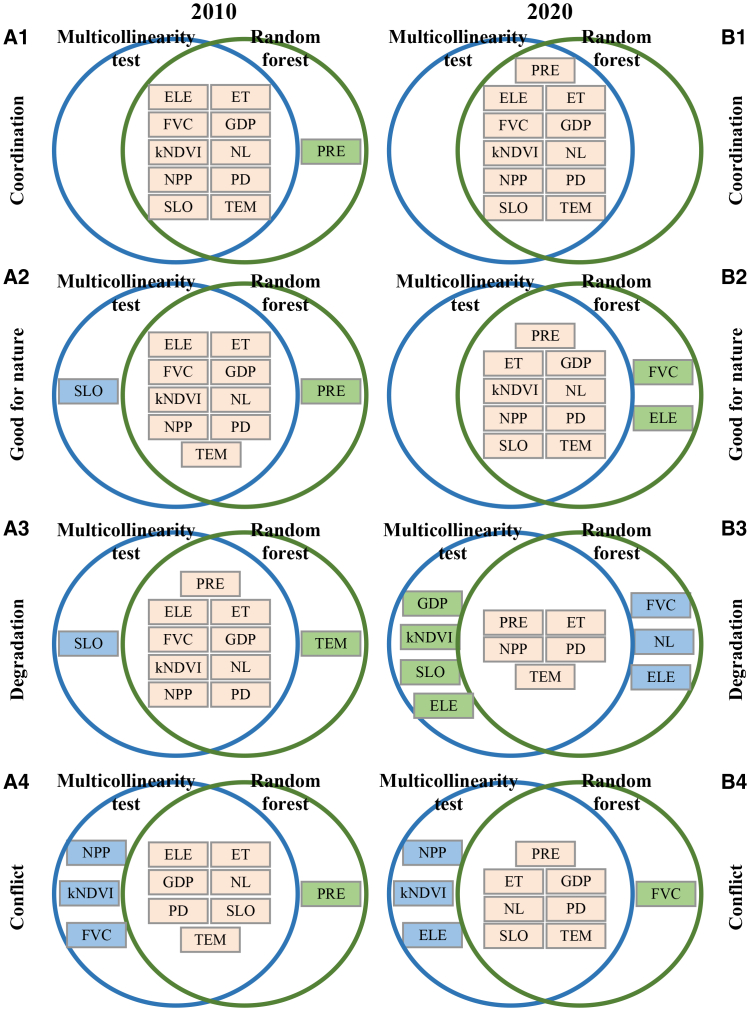
Screening results of influencing factors in four types of regions (A1–A4) 2010. (B1–B4) 2020. Note, the blue and green circles represent the factors screened out using the multicollinearity test and RF model respectively, and their intersection represents the impact factors of CCD after screening.

This study employed multicollinearity tests and the RF model to identify key drivers (see Table S1 and Figure S2 in the supplemental information), with the intersection of factors from both methods considered as the primary determinants of CCD in coordination, conflict, good-for-nature, and degradation regions in 2010 and 2020. As shown in Figure 6, CCD patterns in the coordination and good-for-nature regions were jointly driven by human activities, vegetation dynamics, climate, and topographical conditions. In degradation regions, CCD in 2010 was influenced by all four categories of factors, but by 2020 the dominant drivers narrowed to human activities, vegetation, and climate. In conflict regions, CCD was consistently shaped by human activities, climate, and terrain in both 2010 and 2020.

### Driving mechanisms of coupling coordination degree

**Figure 7:**
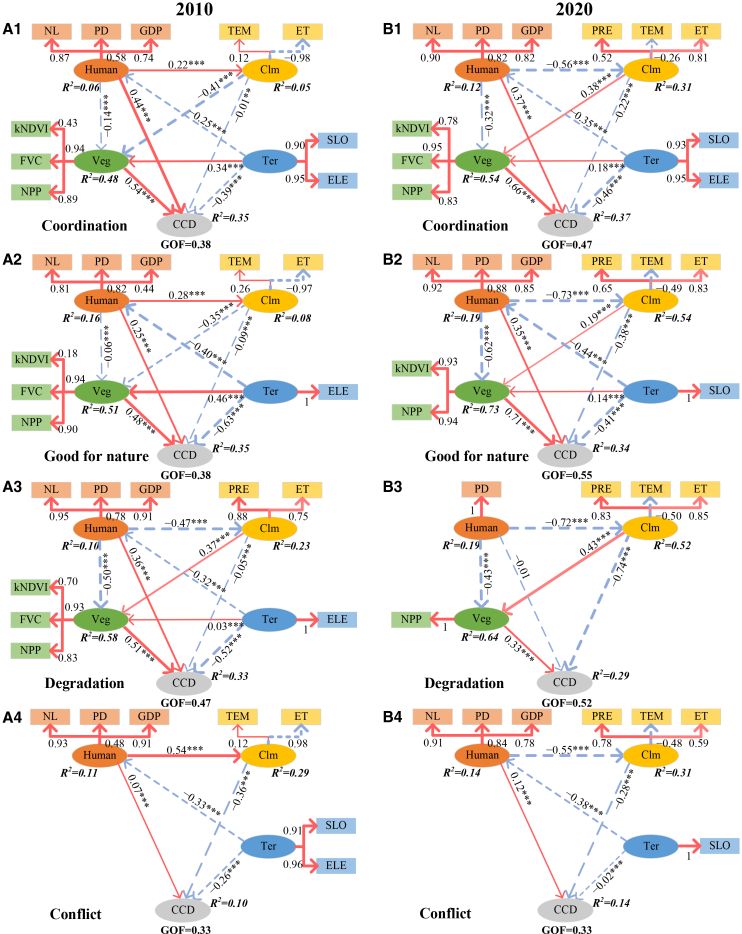
Driving mechanisms of CCD between HFI and EQI in four regions (A1–A4) 2010. (B1–B4) 2020. Note, human, Clm, Veg, and Ter denote human activity, climate, vegetation, and terrain, respectively. The coefficients on the horizontal lines range from −1 to 1. Their signs indicate the direction of influence, and larger absolute values imply greater impacts. ∗∗∗*p* < 0.001, ∗∗*p* < 0.01, ∗*p* < 0.05.

The overall fit of the PLS-SEM was evaluated using the goodness-of-fit (GOF) index, with thresholds of 0.10, 0.25, and 0.36 representing weak, medium, and strong fits, respectively.^41^ As illustrated in Figure 7, except for the conflict region (GOF = 0.33), all models achieved GOF values between 0.38 and 0.55, indicating satisfactory model performance. The PLS-SEM models explained 10%–37% of the variance occurring in 2010 and 2020 across YRDUA.

Figure 7 further illustrates the driving mechanisms of CCD across different regions, revealing marked spatiotemporal heterogeneity. Human activities and vegetation had significant positive effects on CCD during both 2010 and 2020 (path coefficient, *r* = 0.07–0.71, *p* < 0.001), whereas climate and terrain consistently imposed negative effects (*r* = −0.02 to −0.74, *p* < 0.001). An exception occurred in the degradation region in 2020, where the influence of human activity was not significant (*r* = −0.01, *p* > 0.05). Overall, human activity and vegetation are the primary drivers of CCD, while climate and terrain limit its improvement.

At the variable level, nighttime light (NL) emerged as the most stable and dominant indicator of human activity, with factor loadings (*λ*) consistently above 0.8. Population density (PD) and gross domestic product also contributed significantly, though their roles varied across space and time (*λ* = 0.44–1.00). Within the climate group, evapotranspiration was the most influential variable (mean |*λ*| = 0.85), followed by precipitation (0.73) and temperature (0.32). ET generally had positive effects except in degradation regions in 2020. For vegetation, fractional vegetation cover (*λ* = 0.94) and net primary productivity (NPP) (*λ* = 0.90) were the strongest indicators, while kernel normalized difference vegetation index played a comparatively smaller role (*λ* = 0.60). Regarding terrain, both slope (SLO) and elevation (ELE) were highly significant, with loadings above 0.9.

**Figure 8:**
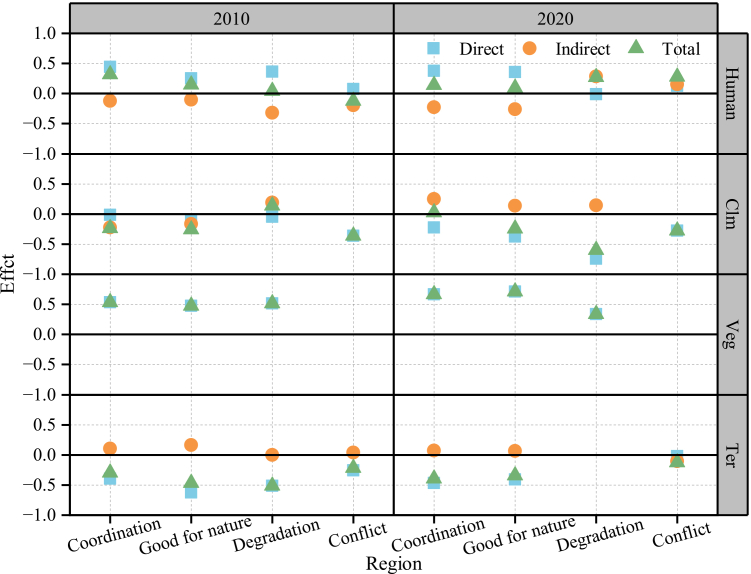
The combined effect of factors Note, human, Clm, Veg, and Ter donate human activity, climate, vegetation, and terrain, respectively. The range of effect values is between −1 and 1, with the sign indicating the direction of the impact. The larger the absolute value of the effect, the greater the influence of the factor.

Human activities also indirectly influenced CCD through climate and vegetation. In 2010, except in degradation areas (*r* = −0.47, *p* < 0.001), human activity had a positive impact on climate across the YRDUA (*r* = 0.22–0.54, *p* < 0.001). By 2020, this relationship turned negative (*r* = −0.55 to −0.72, *p* < 0.001), throughout the YRDUA. Human activity consistently exerted negative effects on vegetation in both 2010 and 2020 (*r* = −0.06 to −0.62, *p* < 0.001). As shown in Figure 8, the indirect effects of human activity on CCD ranged from −0.32 to −0.10 in 2010, with negative effects (−0.26 to −0.23) in coordination and good-for-nature regions. By 2020, these indirect effects became positive (0.15–0.28) in other regions.

Climate also influenced CCD indirectly via vegetation. In 2010, except in degradation areas (*r* = 0.37, *p* < 0.001), climate negatively affected vegetation (*r* = −0.35 to −0.41, *p* < 0.001). By 2020, this relationship turned positive (*r* = 0.19–0.41, *p* < 0.001), and its indirect effects on CCD mirrored these changes, shifting from negative (−0.16 to −0.22) in 2010 to positive (0.13–0.25) in 2020.

Terrain indirectly affected CCD through its influence on human activity and vegetation. Across the YRDUA in both 2010 and 2020, terrain constrained human activity (*r* = −0.25 to −0.44, *p* < 0.001) while supporting vegetation growth (*r* = 0.03–0.46, *p* < 0.001). Except for conflict regions in 2020, the indirect effects of terrain on CCD were consistently positive, ranging from 0.00 to 0.16.

Overall, the total effects of the driving factors on CCD displayed strong spatiotemporal heterogeneity (Figures 7 and 8). Vegetation exerted the strongest positive influence in both 2010 and 2020 (0.33–0.71), although in conflict regions in 2020 human activity became the dominant positive driver (0.28). Climate was the primary negative factor in conflict zones (2010 and 2020) and degradation zones (2020), with effect values ranging from −0.28 to −0.60, whereas terrain was the main negative factor elsewhere (−0.29 to −0.52). The combined influence of other factors was relatively modest, generally within −0.30 to 0.30. These findings underscore the complex and dynamic pathways through which human, climatic, vegetation, and terrain factors jointly shape human-nature coordination, and provide a robust foundation for the interpretations and implications.

## Discussion

### Comparisons of dynamic relationships between the human footprint index and the ecosystem quality index

From 2000 to 2020, the coordination and conflict relationships within human-natural systems in the YRDUA exhibited more pronounced dynamics than those in other regions of China. These differences are largely driven by regional ecosystem background conditions and divergent policy orientations. Approximately 97.6% of the areas in the YRDUA experienced significant changes in both HFI and EQI (Figure 3), a proportion several times higher than that of the Yellow River Basin (44.8%)^25^ and the national average (27.9%).^26^ This highlights the strong imprint of rapid urbanization in the YRDUA on surface systems, resulting in intensified human-nature conflicts. The ratio of coordinated to conflicting zones (3:5) was markedly lower than that observed in the Qinghai-Tibet plateau (5:1),^22^ Hainan province (6:1),^23^ the Yellow river basin (9:1),^25^ and the national average (5:2).^26^ The high coordination of the Tibetan plateau is attributed to its fragile alpine ecosystem and extremely low PD. As a “national ecological security barrier,” this functional positioning dictates that its development must prioritize protection and strictly limit high-intensity exploitation.^22^ Hainan province, leveraging its superior tropical environmental resources, has established a green development path centered on tourism. By implementing measures such as the establishment of national parks, it has restricted industries with high environmental impact at the policy level, fostering a stronger alignment between economic development and ecological protection.^23^ Although the Yellow river basin faces certain pressures, its overall coordination is relatively good, mainly thanks to large-scale ecological projects, such as “returning farmland to forest,” which have improved ecosystem quality in vast non-urban areas like the Loess plateau.^25^ Compared to the aforementioned areas, the human-nature relationship in the YRDUA is characterized by its rapid and high-intensity land development and urbanization process, driven by its role as the national economic engine. The vast urbanized areas, dense population, and economic activities have created intense competition between human footprints and ecological spaces, resulting in a more concentrated and prominent human-environment conflict in this region.

Unlike the quadrant model, which is used to reflect the direction of system evolution, CCD reflects the overall level or quality of the interaction between humans and nature during a specific period. Despite these conflicts, CCD values reveal a more nuanced picture. From 2000 to 2020, over 95% of the YRDUA exhibited CCD values between 0.4 and 0.8, with 51.8%–65.05% falling into the relatively coordinated range of 0.6–0.8 (Figure 4). By comparison, in Hainan province more than 95% of the area exhibited CCD values between 0.2 and 0.6 during the same period, with only a very small proportion achieving values above 0.6.^23^ Therefore, the differences highlighted above reveal two distinct coordination paradigms: the former is characterized by “pressure coordination under high-intensity governance,” where continuous ecological investment supported by a developed economy and advanced ecological management measures maintain the ecosystem at a relatively coordinated level despite intense human activity; the latter is marked by “natural coordination under low-intensity disturbance,” with its lower CCD value reflecting the original balance between limited development and ecological baseline. These findings indicate that the YRDUA model showcases the potential for achieving coordinated development through policy intervention and governance, but it r equires ongoing investment.^26^ In contrast, the Hainan model highlights the cost-effectiveness of source protection, albeit at the cost of limiting development. Thus, these insights are crucial for understanding the sustainable development paths of regions at different stages of growth.

### The drivers of the coupling coordination degree

Based on PLS-SEM analysis, this study finds that human activities in the YRDUA exert an overall positive effect on the CCD of human-natural systems in 2010 and 2020 (Figures 7 and 8). This finding is consistent with previous studies and further deepens our understanding of the coordination mechanisms between human and natural systems in highly developed regions from the perspective of driving pathways. Liu et al.^25^ applied a panel regression model and found that human activity in the Yellow river basin has a significant negative effect on ecosystem quality. Liu et al.^26^ also found that although the increase in human activity has generally supported ecosystem restoration and improvement in China, the rise of HFI in conflict areas still significantly reduces the EQI. Wang et al.^27^ applied an RF model and found that, prior to 2011, the relationship between humans and the natural system in China was mainly driven by natural conditions, whereas after 2011, socio-economic factors became the dominant influence. This study further analyzes the complex mechanisms using the PLS-SEM model: Although human activities exert direct pressure on vegetation (consistent with existing consensus), they also make a net positive contribution to the coupling of humans and nature in most areas of the YRDUA through two pathways—directly enhancing human well-being and indirectly regulating the climate—thus outweighing the negative effects. This not only explains the differences in CCD but also highlights the unique contribution of this study in advancing the understanding of the human-nature coupling driving mechanisms through multi-factor interactions.

The dominant drivers of the CCD exhibited marked spatiotemporal heterogeneity (Figures 7 and 8). Regions with coordination and good-for-nature conditions were shaped by a wider range of impact pathways compared with degradation and conflict regions, reflecting greater sensitivity to both human activities and natural factors. Vegetation resilience in coordinated regions enabled ecosystems to adapt to natural variability, but also made them highly responsive to anthropogenic pressures, resulting in complex and multi-faceted interactions.^38^^,^^42^

The role of topography in degradation regions further exemplifies these dynamics. In 2010, terrain simultaneously restricted human activities and supported vegetation growth, producing a net negative effect on the CCD. By 2020, as degradation regions contracted, the influence of terrain on human activities and vegetation largely offset one another,^38^ resulting in a weakened direct effect. In 2010, degradation regions included both cultivated and forest land, where cultivation intensified human-nature conflicts.^43^ By 2020, these regions had shrunk and were mainly dominated by forest land, with climate emerging as the primary driver of CCD. Under these conditions, human activities influenced CCD indirectly, primarily through their interactions with climate. This shift highlights the increasing dominance of climate-related processes in shaping human-nature coordination under reduced land-use intensity, underscoring the complexity and variability of the driving mechanisms across space and time.

### Policy recommendations

The findings of this study reveal that the coordination between humans and nature within the YRDUA exhibits a polarized development trend, underscoring the necessity for more integrated and targeted interventions. Consequently, this study proposes the following tailored strategies.

In conflict zones (e.g., the Taihu lake basin and the Yangtze river corridor), where core urbanized areas face significant ecological pressures, it is recommended to integrate satellite remote sensing with ground-based observation networks to monitor human activities and the ecological environment in real time. During the infrastructure construction and operation phases, especially for projects along the Yangtze river and around the Taihu lake, it is essential to actively promote nature-based solutions to minimize the impact on the local ecosystem.^44^ In coordination zones (e.g., urban-rural fringes in the northeast), efforts should be made to consolidate and strengthen existing coordination trends by optimizing the ecological spatial network in urban core areas, enhancing ecosystem services through green infrastructure, and exploring the “park city” model.^45^ For good-for-nature and degradation zones (e.g., the forested mountainous areas in southwestern Zhejiang and the cultivated lands in Anhui), the ecological compensation mechanism should be improved, alongside the implementation of targeted restoration projects. Key measures should include restoring water conservation functions and controlling soil erosion in the forested areas of southwestern Zhejiang, enhancing hydrological regulation in Anqing, and repairing soil-vegetation carbon sinks in degraded hotspots like Xuancheng and Chizhou.^46^

### Limitations of the study

This study advances the understanding of human-nature interactions and their complex mechanism in rapidly urbanizing regions by examining the dynamic relationship between HFI and EQI in the YRDUA, offering three key international insights for global sustainable development. At the methodological level, through a grid-scale assessment approach for identifying human-nature relationships and the complex mechanisms of CCD, this study proposes a transferable analytical framework that is applicable to other socio-ecological contexts and valuable for comparative research on human-nature interactions in rapidly urbanizing regions across different continents. At the theoretical level, the study reveals the phenomenon where the YRDUA maintains a high degree of coordination between humans and nature despite the continuous increase in human activity intensity. It provides scientific evidence for the comprehensive positive impact of human activities on the CCD of human-nature systems and uncovers the possibility of achieving a “development-protection” synergy through proactive governance in the context of high-intensity development. This offers important empirical evidence for the Global South regions facing similar development pressures, demonstrating how smart governance can achieve a “win-win” outcome for both development and protection. At the policy practice level, the study reveals the spatial differentiation patterns in the evolution of conflict and coordination, along with the multiple driving mechanisms, offering a model for other countries and regions to implement spatially targeted governance.

However, the analysis is limited by the reliance on decadal data, which may introduce temporal uncertainties, and by the omission of telecoupling effects. As a major global urban agglomeration subject to tourism and cross-regional interactions, the YRDUA experiences external pressures that were not fully captured here. In addition, natural systems are inherently complex systems, and we acknowledge that using the EQI as a proxy offers only a limited viewpoint. Future research could incorporate higher-temporal-resolution data to analyze the driving mechanisms at five-year intervals within the existing regions, or employ dynamic structural equation models to further reveal the long-term evolution of these mechanisms, while explicitly accounting for remote coupling processes. Furthermore, it is expected that subsequent studies will create broader and more accurate metrics for deeper investigation. This would improve the assessment of HFI-EQI dynamics, deepen the understanding of the co-evolution between human pressures and ecological responses, and enhance the generalizability of the framework to diverse regions.

## Resource availability

### Lead contact

Requests for further information and resources should be directed to and will be fulfilled by the lead contact, Ligang Xu (lgxu@niglas.ac.cn).

### Materials availability

This study did not generate new unique reagents.

### Data and code availability

• This study analyzes existing, publicly available data. These accession numbers for the datasets are listed in the key resources table.
• This article does not report original code.
• Any additional information required to reanalyze the data reported in this article is available from the lead contact upon request.

## Acknowledgments

This work was supported by the 10.13039/501100012166National Key Research and Development Program of China (2024YFE0106400, 2023YFF0807204); State Key Laboratory of Lake and Watershed Science for Water Security (2024SKL003); 10.13039/501100001809National Natural Science Foundation of China (U2240224, U2444221); and Jiangxi Science and Technology Program Project (20244BCF61001, 20241ZDD02019, 20223BBG74003, 20243BBH81035).

## Author contributions

Conceptualization, H.Z. and L.X.; methodology, L.G.; investigation, L.G. and Q.Z.; writing – original draft, H.Z.; writing – review and editing, L.X.; funding acquisition, H.Z. and L.X.; formal analysis, H.Z.; data curation, Q.Z.; software, L.G.; visualization, Q.Z. All authors have read and approved the final article.

## Declaration of interests

The authors declare no competing interests.

## STAR★Methods

### Key resources table

**Table:** 

REAGENT or RESOURCE	SOURCE	IDENTIFIER
**Deposited data**		

Human footprint index	Urban Environmental Monitoring and Modeling team	https://www.x-mol.com/groups/li_xuecao/news/48145/
Leaf area index	Earth System Science Data	https://essd.copernicus.org/
Enhanced vegetation index	NASA EarthData	https://www.earthdata.nasa.gov/
Gross primary productivity	National Ecosystem Science Data Center	https://www.nesdc.org.cn/
Precipitation	National Tibetan Plateau/Third Pole Environment Data Center	https://data.tpdc.ac.cn/
Temperature	National Tibetan Plateau/Third Pole Environment Data Center	https://data.tpdc.ac.cn/
Evapotranspiration	NASA EarthData	https://www.earthdata.nasa.gov/
Elevation	Geospatial Data Cloud	https://www.gscloud.cn/
Nighttime light	National Tibetan Plateau/Third Pole Environment Data Center	https://data.tpdc.ac.cn/
Population density	Oak Ridge National Laboratory	https://landscan.ornl.gov/
Gross domestic product	Resource and Environmental Science Data Platform	https://www.resdc.cn/
Net primary productivity	NASA EarthData	https://www.earthdata.nasa.gov/
Surface reflectance	NASA EarthData	https://www.earthdata.nasa.gov/

**Software and algorithms**		

Origin 2022	OriginLab	https://www.originlab.com/
ArcGIS 10.8	Environmental Systems Research Institute	https://www.arcgis.com/
Microsoft Visio 2021	Microsoft	https://www.microsoft.com/
R Project 4.4.2	R Foundation	https://www.r-project.org/

### Experimental model and study participant details

Omitted as our study does not involve biological models.

### Method details

#### Study area

Located on the southeast coast of China, the YRDUA is recognized as one of the world’s six major urban agglomerations (below figure). It comprises Shanghai, nine cities in Zhejiang Province, nine in Jiangsu Province, and eight in Anhui Province, totaling 27 cities. The region is characterized by a subtropical monsoon climate with an annual mean temperature of about 17 °C, and its terrain is dominated by low-lying plains. Although covering only 2.1% of China’s land area, the YRDUA contributes approximately one-quarter of the nation’s economic output and over 20% of its industrial production. This combination of dense urbanization, favorable natural conditions, and exceptional economic importance makes the YRDUA a representative case for examining the human–nature interactions in rapidly urbanizing regions.

**Figure:**
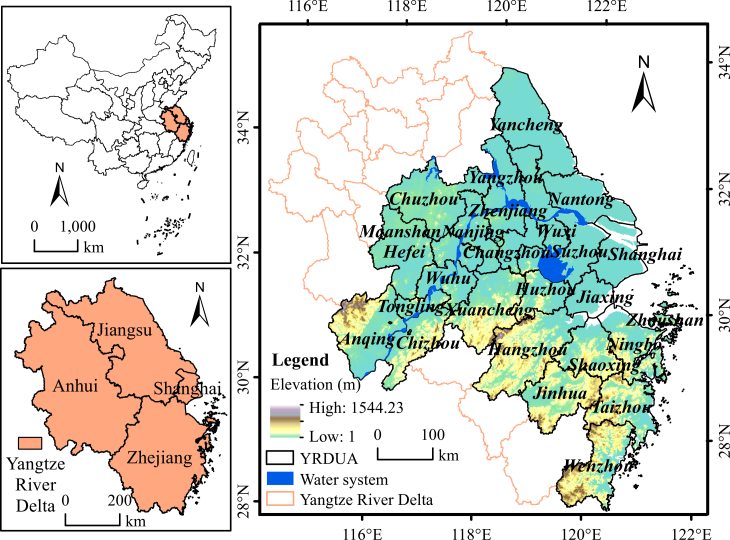
Location of the YRDUA

#### Research framework

The research framework of this study is illustrated in below figure. HFI and EQI are adopted as proxies to represent human and natural systems,^25^^,^^26^ enabling an assessment of their spatiotemporal dynamics in the YRDUA. Building on this foundation, an improved four-quadrant model is applied to identify areas of coordination and conflict between HFI and EQI, while the CCD model is used to quantify the strength of their interactions. To ensure robust identification of influencing factors, the RF model is combined with collinearity diagnostics to screen potential drivers of CCD. Finally, PLS-SEM is employed to uncover the driving mechanisms and to reveal the spatiotemporal heterogeneity of coordination between human and natural systems.

**Figure:**
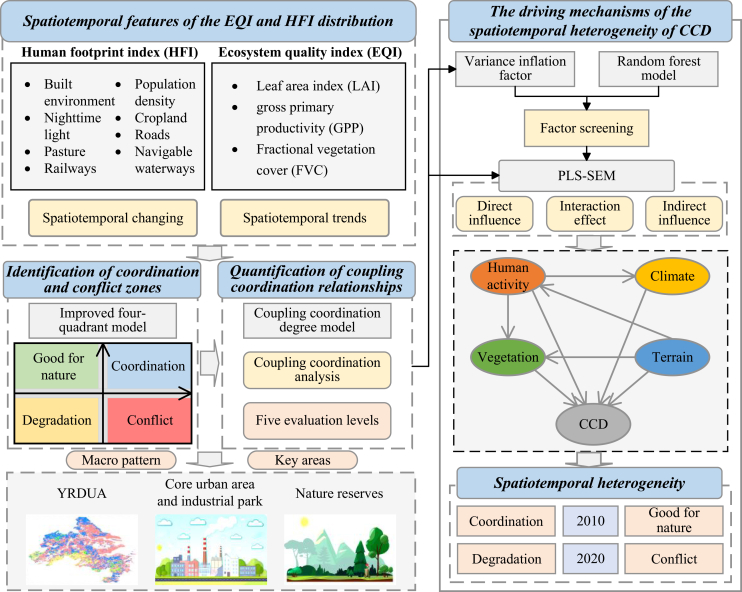
Research framework

#### Data sources

The datasets used in this study cover the period 2000–2020. The HFI data were provided by the Urban Environmental Monitoring and Modeling team (https://www.x-mol.com/groups/li_xuecao/news/48145/) and were used to represent the intensity of human activities.^49^ Leaf area index (LAI) data, processed from MODIS C6.1 products with the STICA algorithm by Yan et al.,^50^ were obtained from the Google Earth Engine (GEE, https://earthengine.google.com/). Enhanced vegetation index (EVI, MOD13A1) data were sourced from NASA EarthData (https://www.earthdata.nasa.gov/), and gross primary productivity (GPP) data were acquired from the National Ecosystem Science Data Center (https://www.nesdc.org.cn/). In addition to these core datasets, four categories of driving factors were considered: climate, terrain, human activities, and vegetation (see below table). Herein, the calculation method for fractional vegetation cover (FVC) is shown in Equation 2. The kernel normalized difference vegetation index (kNDVI) was calculated from the surface reflectance product (MOD09GA V061); the formula and detailed procedures are provided in Camps-Valls et al.^51^ To ensure comparability, all variables were resampled to a spatial resolution of 1 km.

**Table:** Driving factors and their data sources

Categories of drivers	Drivers (abbreviation)	Units (spatial resolution)	Dataset	Sources
Climate	Precipitation (PRE)	mm (1 km)	Peng et al.^47^	https://data.tpdc.ac.cn/
	Temperature (TEM)	°C (1 km)	Peng et al.^47^	https://data.tpdc.ac.cn/
	Evapotranspiration (ET)	mm (500 m)	MOD16A2.061	https://www.earthdata.nasa.gov/
Terrain	Elevation (ELE)	m (90 m)	SRTM	https://www.gscloud.cn/
	Slope (SLO)	° (90 m)	–	Calculated from elevation
Human activity	Nighttime light (NL)	/(1 km)	Zhang et al.^48^	https://data.tpdc.ac.cn/
	Population density (PD)	Per km^2^ (1 km)	LandScan Global Population Data	https://landscan.ornl.gov/
	Gross domestic product (GDP)	CNY 10^4^/km^2^ (1 km)	GDP dataset	https://www.resdc.cn/
Vegetation	Net primary productivity (NPP)	kgC/m^2^/year (500 m)	MOD17A3HGF	https://www.earthdata.nasa.gov/
	Fractional vegetation cover (FVC)	/(500 m)	–	Calculated from Google Earth Engine (GEE, https://earthengine.google.com/)
	Kernel normalized difference vegetation index (kNDVI)	/(500 m)	–	Calculated from GEE

#### Human footprint index

The HFI integrates eight indicators of human activities (See above figure), providing a synthetic measure of anthropogenic pressure on the ecological environment.^49^ It is derived by summing the scores of each indicator, with values ranging from 0 to 50. Compared with land cover or population distribution alone, the HFI offers a more comprehensive proxy for human activity intensity and has been extensively validated and applied across multiple scales, including global, national, basin, and urban levels.^25^^,^^26^^,^^52^ To ensure consistency with the EQI, facilitate spatiotemporal analysis, and provide input for all models in this study, the HFI was normalized to a 0–100 scale using min–max scaling.

#### Ecosystem quality index

The natural system encompasses essential elements such as water, soil, air, and vegetation.^26^ This study focuses on terrestrial ecosystems as a representative of the YRDUA’s natural system, emphasizing human-driven factors that can be derived from accessible grid data. Vegetation quality serves as a core indicator of ecosystem health.^27^^,^^53^ In line with the Technical Specifications for Ecosystem Status Assessment—Ecosystem Quality Assessment (HJ 1172–2021) issued by the Ministry of Ecology and Environment of China, we construct the EQI to assess natural ecosystem conditions. The EQI is widely recognized for its effectiveness in monitoring and evaluating ecosystem health.^25^^,^^26^^,^^54^ This index provides a robust measure of ecosystem health. Other remote sensing indices, such as humidity, heat, and dryness indices,^55^ were not considered, as this study primarily focuses on the interannual impacts of human activities on ecosystem changes. Humidity and heat are largely driven by climate, while the dryness index largely overlaps with the HFI as it reflects urban development. The EQI is formulated:

(Equation 1) $$\begin{array}{c} EQI=\frac{LAI+GPP+FVC}{3}\times 100 \end{array}$$

where *EQI*, *LAI*, *GPP*, and *FVC* represent the ecosystem quality index, leaf area index, gross primary productivity, and fractional vegetation cover, respectively. All three indicators were normalized to a range of 0–1 prior to calculation using min-max scaling method. FVC is calculated from the EVI using the following formula:

(Equation 2) $$\begin{array}{c} FVC=\frac{EVI-{EVI}_{soil}}{{EVI}_{veg}-{EVI}_{soil}} \end{array}$$

where *EVI* denotes the enhanced vegetation index, and *EVI*_*soil*_ and *EVI*_*veg*_ correspond to the *EVI* values of bare soil and fully vegetated areas. To reduce noise and improve robustness, the 5th and 95th percentile *EVI* values were used as proxies for *EVI*_*soil*_ and *EVI*_*veg*_, respectively.^56^^,^^57^

#### Improved four-quadrant model

In this study, a four-quadrant framework was employed to characterize the relationship between HFI and EQI. The interactions between the two indices were classified into four evolutionary types: coordination, good for nature, degradation, and conflict. To distinguish significant from minor variations, regions with changes in HFI and EQI smaller than 10% of their respective standard deviations during 2000–2010 and 2010–2020 were defined as insignificant.^22^^,^^27^

As shown in below figure, the first quadrant represents coordinated evolution, where both HFI and EQI increase, indicating the co-prosperity of human society and the natural ecosystem. The second quadrant denotes reduced human activity, often driven by policy interventions or technological advancements, accompanied by improved ecosystem quality (“good for nature”). The third quadrant reflects simultaneous declines in both HFI and EQI, potentially linked to climate change or policy-related challenges (“degradation”). The fourth quadrant illustrates conflict, where human activity intensifies while ecosystem quality declines. The meanings of the above four-quadrant divisions are consistent with previous studies.^22^^,^^26^^,^^27^ This framework provides a straightforward approach to capture divergent human–nature trajectories across space and time, offering valuable insights into regional sustainability pathways.

**Figure:**
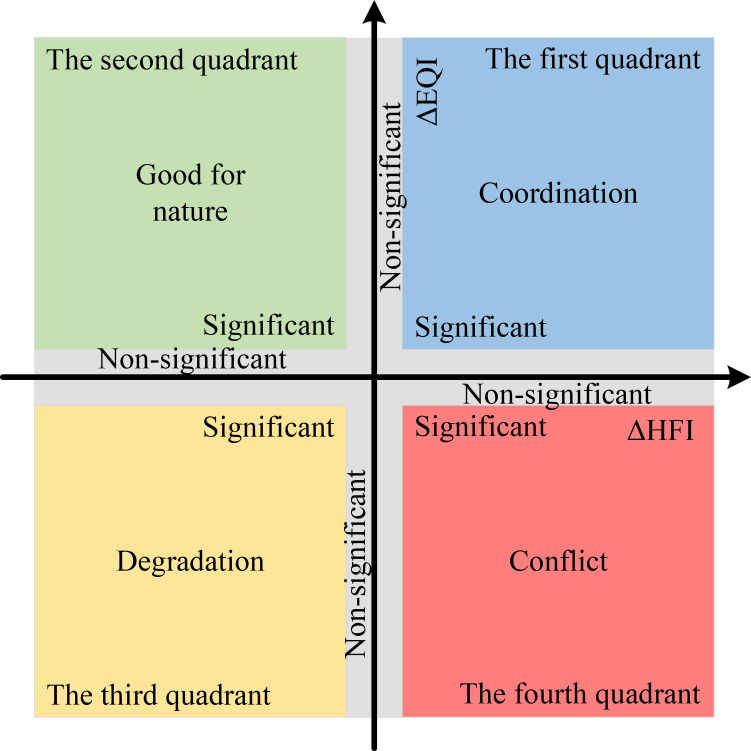
Four-quadrant diagram of human-nature relationships

#### Coupling coordination degree model

The CCD model is widely used to evaluate the interaction state between two or more systems.^14^ In this study, the CCD model was used to quantify the coupling coordination relationship between the HFI and the EQI. First, the HFI values normalized to 0–100 previously and the EQI values were rescaled to 0–1 using min–max normalization, as required by the CCD model formulation. Then, the CCD is calculated using the following formula:

(Equation 3) $$\begin{array}{c} C=2\times \frac{\sqrt{HFI\times EQI}}{HFI+EQI} \end{array}$$

(Equation 4) $$\begin{array}{c} T=aHFI+bEQI \end{array}$$

(Equation 5) $$\begin{array}{c} CCD=\sqrt{C\times T} \end{array}$$

where *C* represents the coupling degree; *T* reflects the comprehensive level of HFI and EQI. Parameters *a* and *b* denote the relative contributions of HFI and EQI to the coupled system. Following Lei et al.,^23^ Han et al.,^58^ Ge et al.,^59^ and Fang et al.,^60^ who argue that human and natural systems are equally important for sustainable development, both *a* and *b* were set to 0.5 in this study. The resulting CCD values were classified into five coordination categories (see below table), following established criteria.^23^^,^^61^

**Table:** The levels of coupling coordination degree

CCD types	Highly coordinated	Moderately coordinated	Primarily coordinated	Nearly disordered	Severely disordered
CCD	0.8 ≤ CCD ≤1	0.6 ≤ CCD ≤0.8	0.4 ≤ CCD ≤0.6	0.2 ≤ CCD ≤0.4	0 ≤ CCD ≤0.2

#### Impact factors screening

Climate, terrain, human activities, and vegetation were considered as the primary factors influencing the HFI, EQI, and their CCD. Based on previous studies,^23^^,^^26^^,^^38^ eleven representative variables were selected as potential driving factors (see above table).

RF, a widely used ensemble learning algorithm, was applied to evaluate the relative importance of these variables. RF constructs multiple decision trees by randomly sampling both training data and predictor subsets, offering high stability and predictive accuracy. In this study, an ensemble of 500 trees was used to compute the permutation importance scores (%IncMSE) for candidate predictors. Statistical significance tests were also incorporated to refine the ranking of predictor importance.

Although RF model is generally robust to multicollinearity, highly correlated variables can hinder interpretability. To address this, the variance inflation factor (VIF) was employed to test for collinearity, and all predictors with VIF values above 6 were excluded.^62^

#### Partial least squares structural equation modeling

PLS-SEM was employed to explore the causal networks among latent variables and to quantify the complex interrelationships reflected by their associated observed indicators. The model consists of two components: a measurement model and a structural model.^63^

The measurement model links latent variables to their observed indicators,^64^ and can be expressed as:

(Equation 6) $$\begin{array}{c} X=\lambda_{x}\xi +\varepsilon \end{array}$$

(Equation 7) $$\begin{array}{c} Y=\lambda_{y}\eta +\varepsilon \end{array}$$

where *X* and *Y* represent exogenous indicators and endogenous indicators, respectively; *λ*_*x*_ and *λ*_*y*_ denote the factor loading matrices of exogenous latent variables *ξ* on *x* and endogenous latent variables *η* on *y*, respectively; and *ε* represents the measurement error.

The structural model delineates the relational pathways among latent variables.^64^ The formulation of this model is as follows:

(Equation 8) $$\begin{array}{c} \eta =A\eta +r\xi +\zeta \end{array}$$

where *A* denotes the influence of exogenous latent variables on endogenous latent variables; *r* represents the path coefficients between latent variables; and *ζ* is the regression residual.

Based on the validated pathways and the definition of the coupling between human and natural systems,^23^^,^^26^^,^^38^^,^^65^^,^^66^ the following hypotheses were tested within the PLS-SEM framework: (1) Human activities, climate, vegetation, and terrain directly influence CCD; (2) Human activities, climate, and terrain indirectly affect CCD through vegetation; (3) Human activities indirectly influence CCD through climate; (4) Terrain indirectly influences CCD through human activities.

### Quantification and statistical analysis

Statistical analyses were performed using R 4.4.2 and ArcGIS 10.8. All statistical details, including the specific tests used and significance levels, are reported in the main-text and Figures 1, 2, 3, 4, 5, 6, 7, and 8. The “Slope” function was used to calculate the slope in ArcGIS 10.8. The RF algorithm and PLS-SEM were implemented with the “randomForest” and the “plspm” packages in R 4.4.2, respectively. Statistical significance was defined as ∗∗∗*p* < 0.001, ∗∗*p* < 0.01, ∗*p* < 0.05.
