# Supplementary material for: Unveiling the influence pathways of the evolution of human-nature relationships in the Yangtze river delta urban agglomeration
Source: iScience. 2026 Feb 10;29(3):114989. doi: 10.1016/j.isci.2026.114989 (PMC12962159; doi:10.1016/j.isci.2026.114989)
Supplement: Document S1. Figures S1 and S2 and Table S1 [file mmc1.pdf]

iScience, Volume *29*

Supplemental information

Unveiling the influence pathways of the evolution of human-nature relationships in the Yangtze river delta urban agglomeration

Hua Zhu, Liang Gan, Qing Zhang, and Ligang Xu

Supplemental information

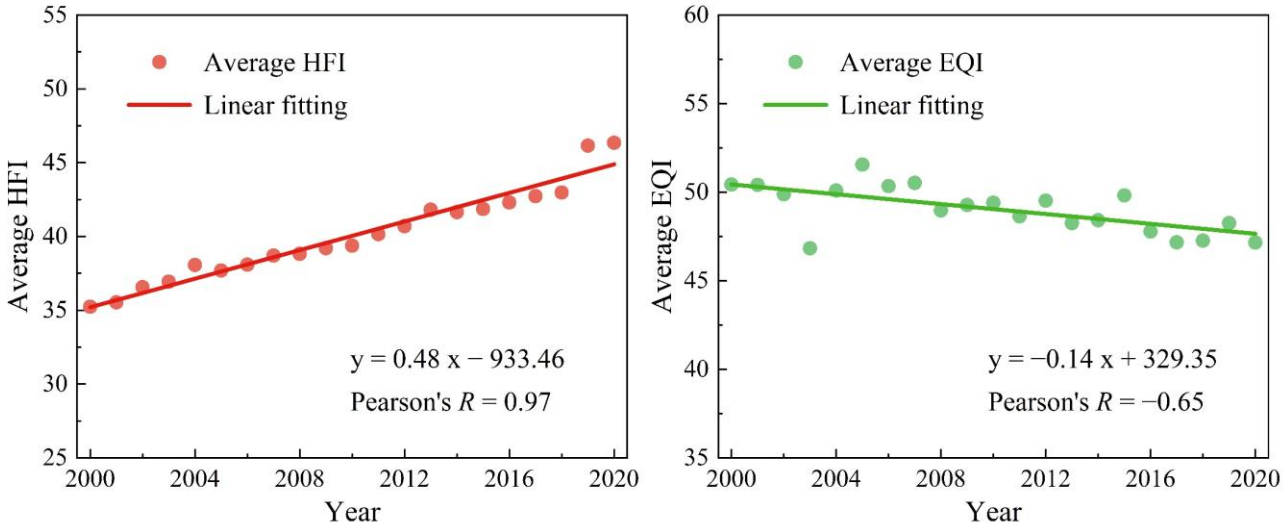

Figure S1. Temporal trend of HFI and EQI

Table S1. Variance inflation factor after factor screening

Variable	2010				2020			
	1	2	3	4	1	2	3	4
ELE	2.58	3.94	2.66	2.63	4.25	/	/	5.09
ET	1.69	1.50	1.59	2.25	2.04	2.56	3.31	2.08
FVC	3.15	3.00	3.89	/	4.88	/	/	/
GDP	2.12	1.52	3.82	2.97	1.82	2.13	1.77	1.71
kNDVI	1.70	1.39	2.62	2.56	2.51	4.14	4.52	1.83
NL	2.27	1.74	5.11	3.49	2.42	4.32	/	2.45
NPP	2.57	2.42	2.06	3.24	2.72	3.27	3.71	2.44
PD	1.10	1.26	1.65	1.12	1.69	2.54	3.30	1.91
SLO	2.78	2.87	2.18	3.01	3.21	2.23	2.41	3.25
PRE	/	/	2.98	/	2.28	1.71	2.02	2.30
TEM	1.42	1.67	/	1.36	1.85	1.28	1.39	1.96

Note: The numbers 1, 2, 3, and 4 represent coordination, good-for-nature, degradation, and conflict areas, respectively.

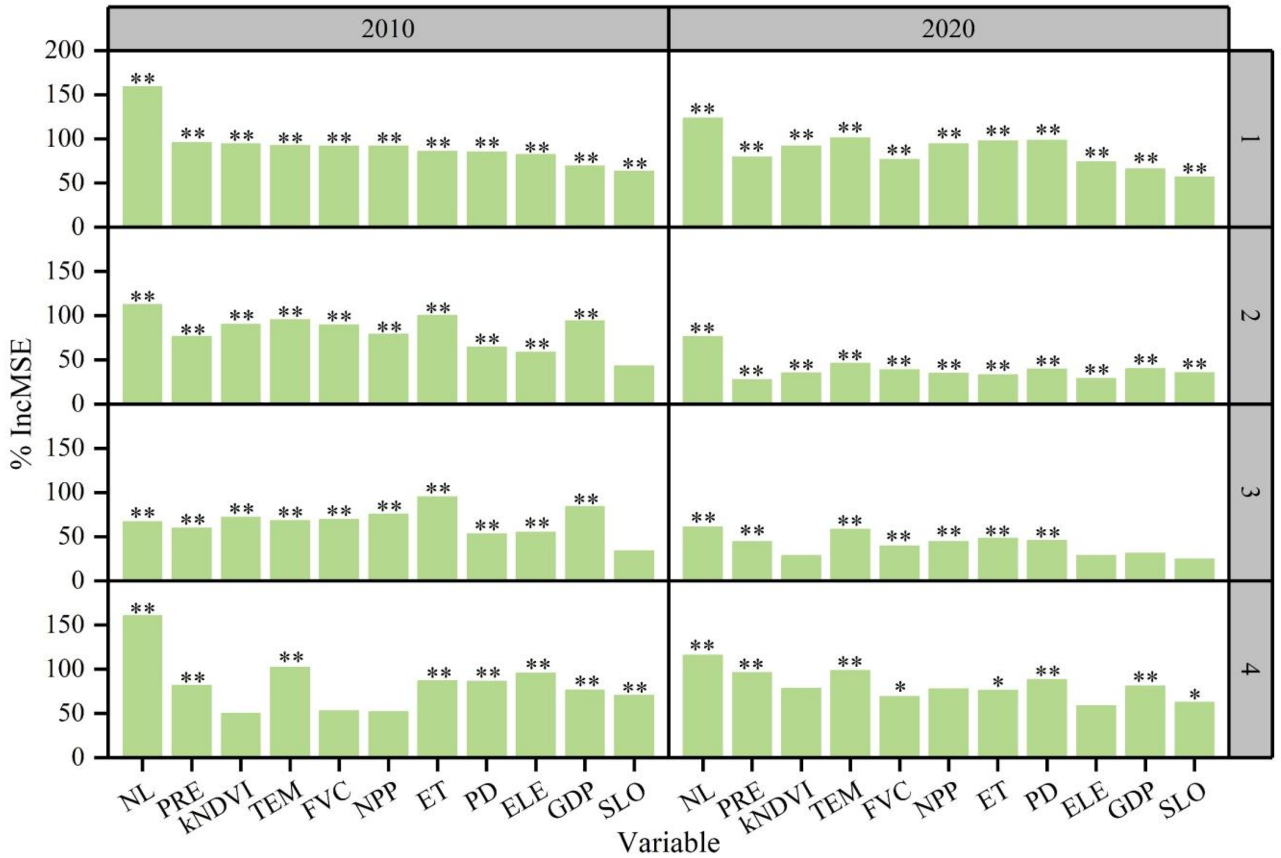

Figure S2. Permutation importance score of variables

Note: The numbers 1, 2, 3, and 4 represent coordination, good-for-nature, degradation, and conflict areas, respectively. $**p < 0.01$, $*p < 0.05$.
